# *In Vitro* and *In Vivo* Leishmanicidal Activity of Beauvericin

**DOI:** 10.1021/acs.jnatprod.4c01098

**Published:** 2024-12-03

**Authors:** Virlânio A. de Oliveira Filho, Juliana R. Gubiani, Vitória D. Borgonovi, Felipe Hilário, Marcelo R. de Amorim, Karen Minori, Vitor K. S. Bertolini, Antonio G. Ferreira, Andressa R. Biz, Marcos A. Soares, Helder L. Teles, Fernanda R. Gadelha, Roberto G. S. Berlinck, Danilo C. Miguel

**Affiliations:** † Instituto de Biologia, Universidade Estadual de Campinas, Campinas 13083-862, SP, Brazil; ‡ Instituto de Química de São Carlos, Universidade de São Paulo, CP 780, 13560-970 São Carlos, SP, Brazil; ⊥ Departamento de Química, Universidade Federal de São Carlos, São Carlos 13565-905, SP, Brazil; ∇ Departamento de Botânica e Ecologia. Universidade Federal de Mato Grosso − UFMT, Cuiabá 78060-900, MT, Brazil; § Instituto de Ciências Exatas e Naturais, Universidade Federal de Rondonópolis, Campus de Rondonópolis, 78736-900 Rondonópolis, MT, Brazil

## Abstract

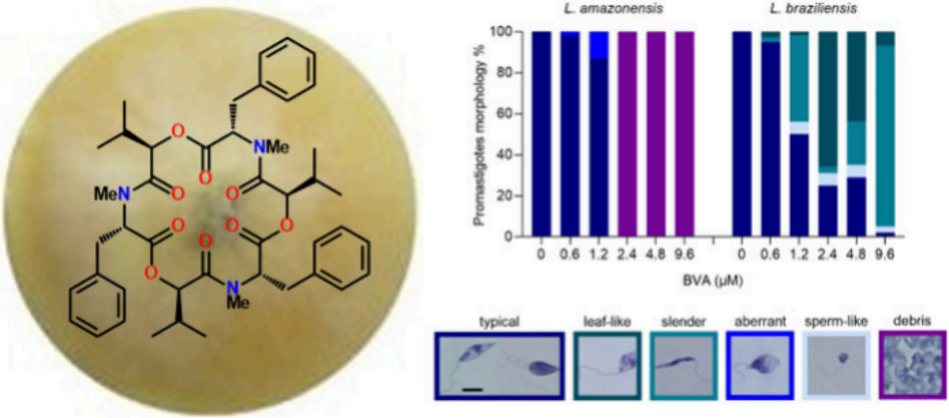

Leishmaniasis is a worldwide disease caused by more than 20 species of *Leishmania* parasites. *Leishmania amazonensis* and *L. braziliensis* are among the main causative agents of cutaneous leishmaniasis, presenting a broad spectrum of clinical forms. As these pathologies lead to unsatisfactory treatment outcomes, the discovery of alternative chemotherapeutic options is urgently required. In this investigation, a leishmanicidal bioassay-guided fractionation of the growth media extract produced by *Aspergillus terreus* P63 led to the isolation of the cyclic depsipeptide beauvericin (**1**). The viability of *L. amazonensis*, *L. braziliensis* and mammalian cells (macrophages and L929 fibroblasts) was assessed in **1** incubated cultures. *Leishmania* promastigotes were sensitive to **1**, with EC_50_ values ranging from 0.7 to 1.3 μM. Microscopy analysis indicated that *Leishmania* spp. parasites showed morphological abnormalities in a dose-dependent manner in the presence of **1**. *L. amazonensis* intracellular amastigotes were more sensitive to **1** than promastigotes (EC_50_ = 0.8 ± 0.1 μM), with a good selectivity index (22–30). **1** reduced the infectivity index at very low concentrations, maintaining the integrity of the primary murine host cell for up to the highest concentration tested for **1**. *In vivo* assays of **1** conducted using BALB/c mice infected with stationary-phase promastigotes of *L. amazonensis* in the tail base presented a significant reduction in the lesion parasite load. A second round of *in vivo* assays was performed to assess the efficacy of the topical use of **1**. The results demonstrated a significant decrease in the total ulcerated area of mice treated with **1** when compared with untreated animals. Our results present promising *in vitro* and *in vivo* leishmanicidal effects of beauvericin, emphasizing that systemic inoculation of **1** led to a decrease in the parasite load at the lesion site, whereas topical administration of **1** delayed the progression of leishmaniasis ulcers, a cure criterion established for cutaneous leishmaniasis management.

Neglected Tropical Diseases (NTDs) constitute a group of 20 different pathologies found in tropical and subtropical regions affecting mainly economically disadvantaged communities. NTDs lead to serious health, social, and economic outcomes for over one billion people.^1^ With more than 1 million new cases annually, vector-borne NTD leishmaniasis presents high mortality and morbidity rates. Its etiologic agents are protozoan parasites that belong to the genus *Leishmania* and are transmitted during the blood meal of infected female phlebotomines. There are two main stages in *Leishmania*’s life cycle: the extracellular flagellated forms known as promastigotes, found parasitizing the digestive tract of the insects, and the amastigote stage that resides within cells of the mononuclear phagocytic system of the vertebrate host, preferably macrophages.^2,3^

Leishmaniasis is characterized by its visceral and/or cutaneous symptoms, which can be explained by the infecting species, endemic area, and the host’s immune response. Clinical manifestations of leishmaniasis are described as cutaneous localized leishmaniasis (CL) characterized by erythematous papules that develop as ulcers with elevated borders; disseminated leishmaniasis (DL), defined by acneiform eruptions, mainly on the trunk and face; mucosal leishmaniasis (ML), affecting the nasal and/or oral mucosa that may reach pharynx; and the diffuse clinical form (DCL), which is associated with the host poor cellular immune response, leading to the progression of nonulcerated nodules throughout the body. As a result, the skin lesions on exposed areas can lead to lifelong scars representing a social stigma. ML and DCL are primarily caused by *L. braziliensis* and *L. amazonensis* in the Americas, respectively. The visceral form of the disease (VL), found in the Old and New World, is characterized by a systemic infection causing fever, anemia, and hepatosplenomegaly that if misdiagnosed and untreated results in death.^4,5^

Chemotherapy available for leishmaniasis treatment, so far restricted to pentavalent antimonials, amphotericin B, pentamidine and paromomycin, presents relevant bottlenecks, such as severe side effects, parenteral administration for most of the options, and reports of resistant *Leishmania* strains.^5,6^ Miltefosine emerged in the past decades as an option against VL, but its severe gastrointestinal effects and teratogenic potential have limited its use. There are no vaccines or prophylactic drugs for the treatment of leishmaniasis. In this scenario, the search for more effective treatments capable of generating fewer side effects and topical administration in the case of cutaneous forms, is in urgent need.^5,6^

Natural products, especially plant- and fungal-based products, have long been used in traditional medicine. Natural products have been extensively investigated as antileishmanial agents; however, very few natural products have been evaluated as *in vivo* antiparasitic agents.^7−12^

*Aspergillus terreus* is a fungal species that has been extensively investigated. Over 172 bioactive secondary metabolites have been reported as produced by *A. terreus* as of 2022, including beauvericin (**1**), the cholesterol-lowering drug lovastatin, the antitumor butyrolactone I and the antiviral acetylaranotin.^13−16^ In this investigation, we describe the bioassay-guided isolation of **1** from culture media of *A. terreus* P63 and its *in vitro* and *in vivo* leishmanicidal activity on *Leishmania* species, causing cutaneous manifestations of leishmaniasis in the Americas.
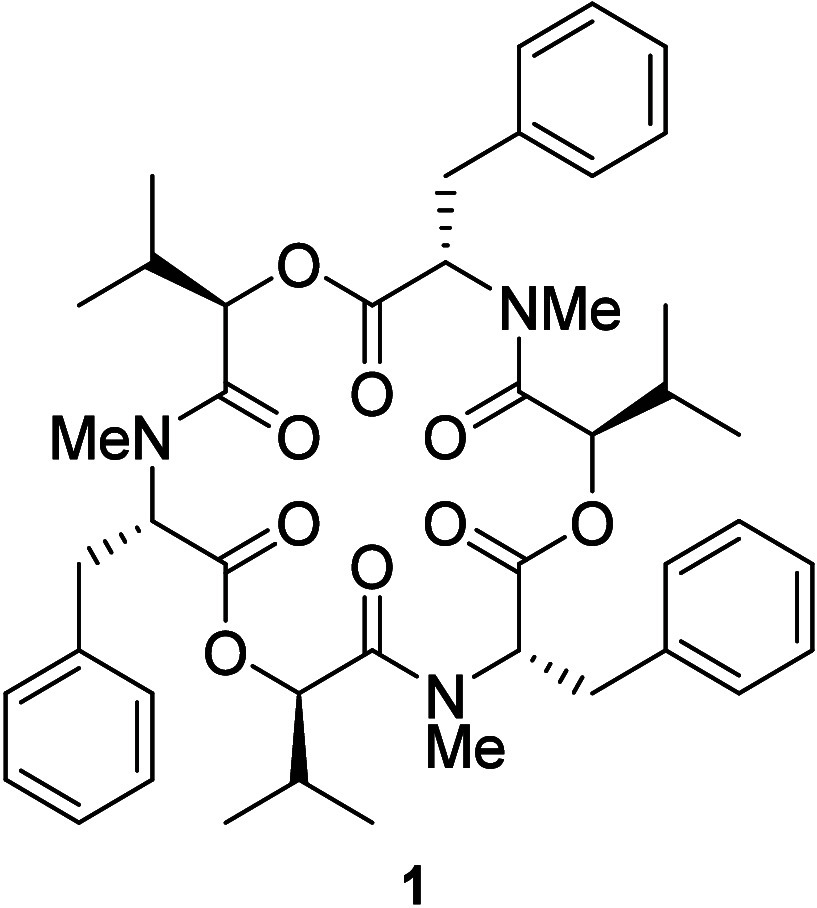


## Results and Discussion

The cyclic hexadepsipeptide **1** was isolated in large amounts (almost 1.0 g) from the organic-soluble extract obtained from the growth medium produced by *A. terreus* P63. This fungal strain was isolated from the roots of the grass *Axonopus leptostachyus. A. terreus* P63 was cultured on rolled oats for 30 days. After growth, the cultures were extracted with MeOH, and the MeOH extract was concentrated and partitioned with hexane. The MeOH fraction was evaporated to dryness, suspended in EtOAc/H_2_O 1:1 and subjected to a liquid–liquid partitioning. The EtOAc fraction was subjected to a bioassay-guided fractionation to yield **1** (934.0 mg), identified by analysis of spectroscopic data. Beauvericin (**1**) was isolated as a yellowish amorphous optically active solid, with [α]_D_^23^ + 65.5 (*c* 1.0, MeOH) [literature: [α]_D_^23^ + 65.8 (*c* 1.0, MeOH)].^17^ The purity of **1** was evaluated as >98% by ^1^H NMR analysis (Figures S2–S4, Supporting Information).

**1** was effective in killing all *Leishmania* spp. parasites in the low micromolar range concentration after 24 h (Table 1). **1** was also tested on *Trypanosoma cruzi* epimastigotes, a *Leishmania*-related parasite, and presented an EC_50_ in the same range as for *Leishmania* spp. parasites. In parallel, the activity of **1** on primary macrophages was evaluated to determine its cytotoxicity potential for a classic *Leishmania*-host cell model (BMDM). The CC_50_ observed was higher than the *Leishmania* EC_50_, providing good selectivity indexes (SI) for **1** (from 14.8 to 29.7; Table 1). An assay on L929 fibroblasts, host cells capable of sustaining *T. cruzi* infections, provided results that represented even more resistance to **1** (45.5 ± 0.9 μM). In this case, the selective activity of **1** was close to the one obtained for BMDM/*Leishmania* promastigotes (SI = 13.4).

**Table 1:** *In Vitro* Activity of Beauvericin (**1**) on *L. amazonensis*, *L. braziliensis*, *T. cruzi*, L929 Fibroblasts, and Bone Marrow-Derived Macrophages^a^

Parasite or host cell/Stage	EC_50_ or CC_50_ ± SD (μM)^b^	Selectivity index (SI)
*L. amazonensis*/promastigote	1.2 ± 0.1	14.8
*L. amazonensis*/intracellular amastigote	0.8 ± 0.1	22.3
*L. braziliensis*/promastigote	0.9 ± 0.2	19.8
*L. braziliensis*/intracellular amastigote	0.6 ± 0.1	29.7
Bone marrow-derived macrophages (BMDM)	17.8 ± 0.4	–
*T. cruzi*/epimastigote	3.4 ± 0.8	13.4
L929 fibroblasts	45.5 ± 0.9	–

a Cell viability, measured by the MTT assay, was determined after exposure to different concentrations of **1** for 24 h.

b (EC_50_): 50% effective concentration. (CC_50_): 50% cytotoxicity concentration. (SD): standard deviation. (SI): BMDM CC_50_/*Leishmania* EC_50_ and L929 CC_50_/*T. cruzi* EC_50_. (−): not applicable.

In parallel, drugs routinely used in the therapy of leishmaniasis (amphotericin B) and Chagas disease (benznidazole) were assayed against the axenic forms of the parasites. The results obtained indicated EC_50_ values in the range between 0.2 and 0.64 μM for *Leishmania* spp. and in 2.23 ± 0.1 μM range for *T. cruzi*. The activity values obtained for **1** are in the same concentration range observed for the control drugs.

**Figure 1:**
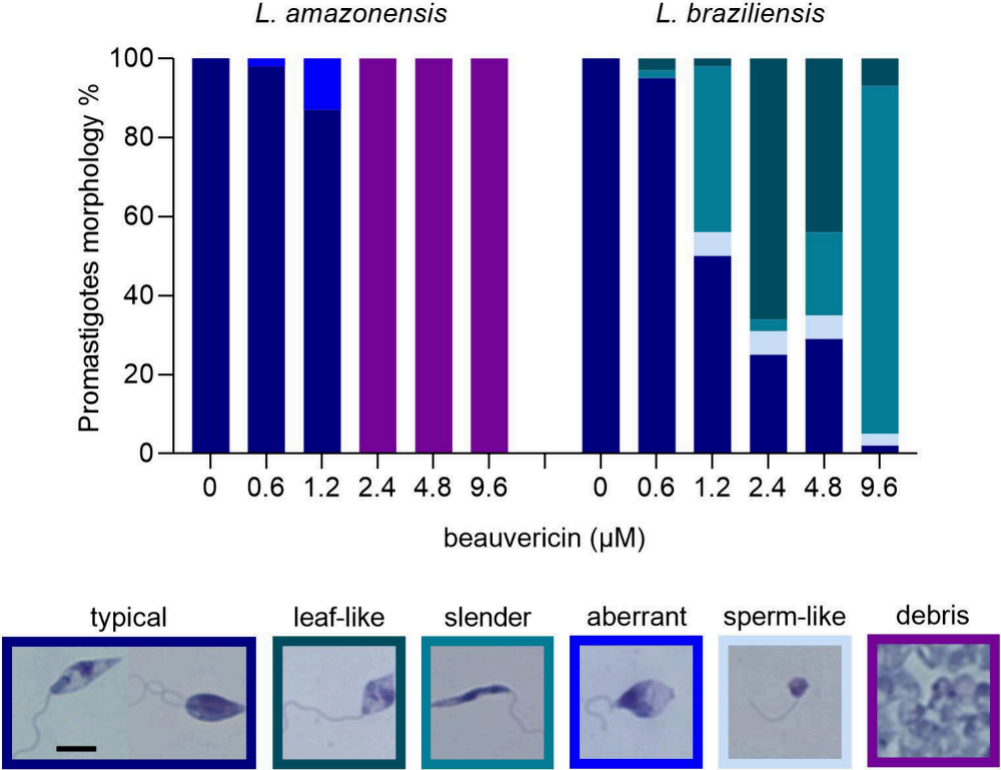
Morphological aspects of *L. amazonensis* and *L. braziliensis* promastigotes upon 24 h incubation with beauvericin (**1**). Corresponding aliquots of **1** concentrations (0, 0.6, 1.2, 2.4, 4.8, and 9.6 μM) tested in the viability assay were fixed and stained on glass slides, and photographs were taken for quantification of promastigote morphotypes. At least 200 cells were counted for each condition. Six representative images are shown featuring each morphotype found: *typical* (fusiform and replicant), *leaf-like*, *slender*, *aberrant*, *sperm-like* and *debris*. The color of each image border corresponds to the frequency of the colored bars in the graph. Scale bar: 5 μm.

Next, we assessed promastigote morphology in thick smear slides to detect possible cellular alterations of incubated parasites in the presence of **1** (Figure 1). Control cultures of *L. amazonensis* and *L. braziliensis* were fully composed by classical individual fusiform or replicating promastigotes (“typical”). When *L. amazonensis* parasites were incubated with increasing concentrations of **1**, the cultures exhibited morphological changes up to 1.2 μM, that included shortening and rounding of the cell body, with kinetoplastid DNA (kDNA) and nuclei not well distinguished (“aberrant”), and massive cell “debris” formation at 2.4, 4.8, and 9.6 μM.

*L. braziliensis* promastigotes, on the other hand, showed more pronounced morphological alterations when exposed to **1**, in concentrations from 1.2 to 9.6 μM, that included the occurrence of “slender” cell bodies, “sperm-like” structures and “leaf-like” promastigotes, for which kDNA and nuclei were still preserved after staining (Figure 1).

In addition to the characterization of the direct effect of beauvericin on promastigotes, we aimed to investigate whether **1** would be able to reduce the intracellular parasite burden in infected BMDM. Propelled by the good SI obtained from BMDM CC_50_ and *Leishmania* promastigotes EC_50_s (>14.8; Table 1), we examined the activity of **1** against intracellular amastigotes.

**Figure 2:**
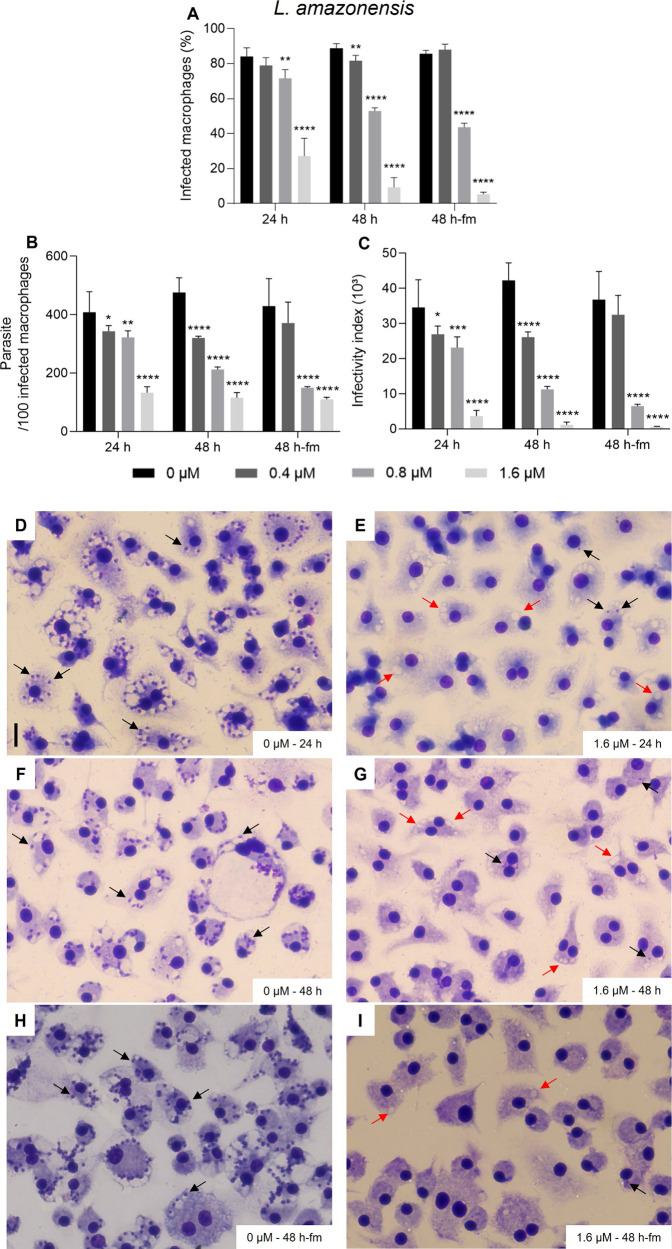
Activity of beauvericin (**1**) on BMDM infected with *L. amazonensis. In vitro* infections were performed using a multiplicity of infection (MOI) = 5 and maintained in fresh culture medium with 0, 0.4, 0.8, and 1.6 μm of BVA for “24 h”, “48 h” or 24 h plus 24 h with **1** and addition of fresh medium (“48 h-fm”). To determine the extent of intracellular infections (A), parasite load (B) and infectivity index (multiplication of A and B), at least 300 host cells were counted per infection and calculations were made in relation to untreated infection groups (“0”), for two independent assays performed in triplicates. * *p* < 0.05, ** *p* < 0.01, *** *p* < 0.001, **** *p* < 0.0001. Representative images of the untreated group for 24 h (D), 48 h (F) and 48 h-fm (H) are shown. Representative images of cultures incubated with 1.6 μM of **1** for 24 h (E), 48 h (G) and 48 h-fm (I). Cells were fixed in MeOH, stained with Instant Prov Kit (NewProv) and images were obtained using EVOS XL Core Cell Imaging System. Scale bar = 20 μm. Black arrows point to intracellular amastigotes. Red arrows point to vacuoles lacking amastigotes.

**Figure 3:**
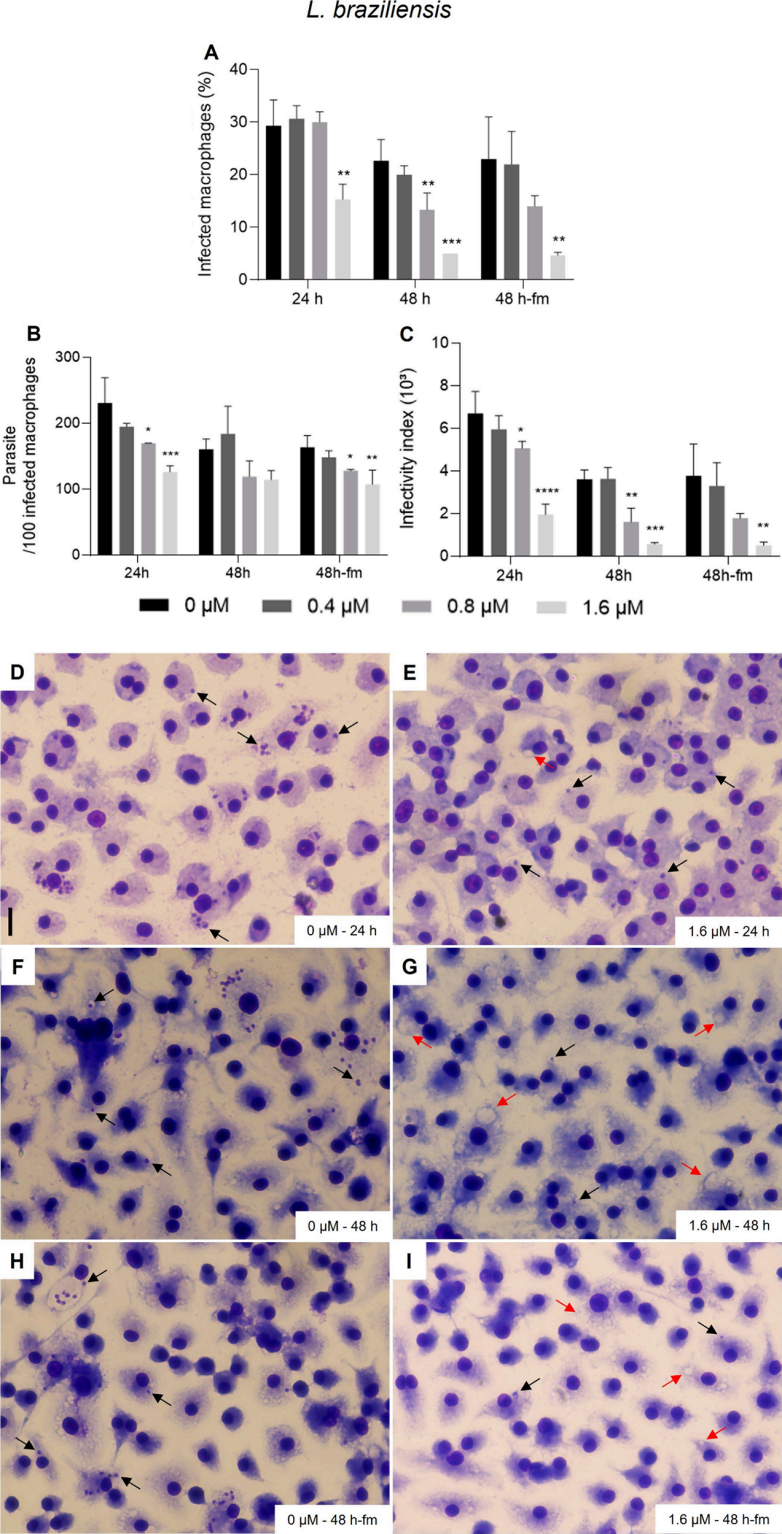
Activity of beauvericin (**1**) on BMDM infected with *L. braziliensis. In vitro* infections were performed in triplicate using a multiplicity of infection (MOI) = 10 and maintained in fresh culture medium with 0, 0.4, 0.8, and 1.6 μm of **1** for “24 h”, “48 h” or 24 h plus 24 h with **1** and addition of fresh medium (“48 h-fm”). To determine the extent of intracellular infections (A), parasite load (B) and infectivity index (multiplication of A and B), at least 300 host cells were counted per infection and calculations were made in relation to untreated infection groups (“0”), for two independent assays performed in triplicates. * *p* < 0.05, ** *p* < 0.01, *** *p* < 0.001, **** *p* < 0.0001. Representative images of the untreated group for 24 h (D), 48 h (F) and 48 h-fm (H) are shown. Representative images of cultures incubated with 1.6 μM of **1** for 24 h (E), 48 h (G) and 48 h-fm (I). Cells were fixed in MeOH and stained with Instant Prov Kit (NewProv), and images were obtained using EVOS XL Core Cell Imaging System. Scale bar: 20 μm. Black arrows point to intracellular amastigotes. Red arrows point to vacuoles lacking amastigotes.

*In vitro* infections were incubated with beauvericin (**1**) at 0, 0.4, 0.8, and 1.6 μM for three time points: “24 h”, “48 h” or 24 h plus 24 h with **1** and fresh culture medium addition (“48 h-fm”; Figures 2 and 3). The most significant extent of inhibition was observed especially at 48 h, with no dependence on additional dosage of **1** in fresh medium (Figure 2). The infectivity index (infection rate *x* parasite burden) for *L. amazonensis* decreased remarkably with incubation of beauvericin (**1**) at 1.6 μM (*ca*. 95% reduction relative to that of untreated control infections). Healthy infected BMDM incubated with **1** were observed showing vacuoles lacking amastigotes (red arrows; Figure 2G). *L. braziliensis* intracellular amastigotes proved to be similarly sensitive to **1** as seen for *L. amazonensis*, with 89% reduction of the infectivity index for cultures incubated with 1.6 μM for 48 h (Figure 3). Also in this case, the highest concentration of **1** did not lead to cytotoxicity alterations in BMDM (Figure 3G).

After establishing the *in vitro* effect of beauvericin (**1**) against intracellular amastigotes, we conducted a set of *in vivo* experiments using *L. amazonensis* and BALB/c mice; as for this murine model of infection, cutaneous lesions develop gradually, making it possible to follow up the effect of a given antileishmanial candidate.

**Figure 4:**
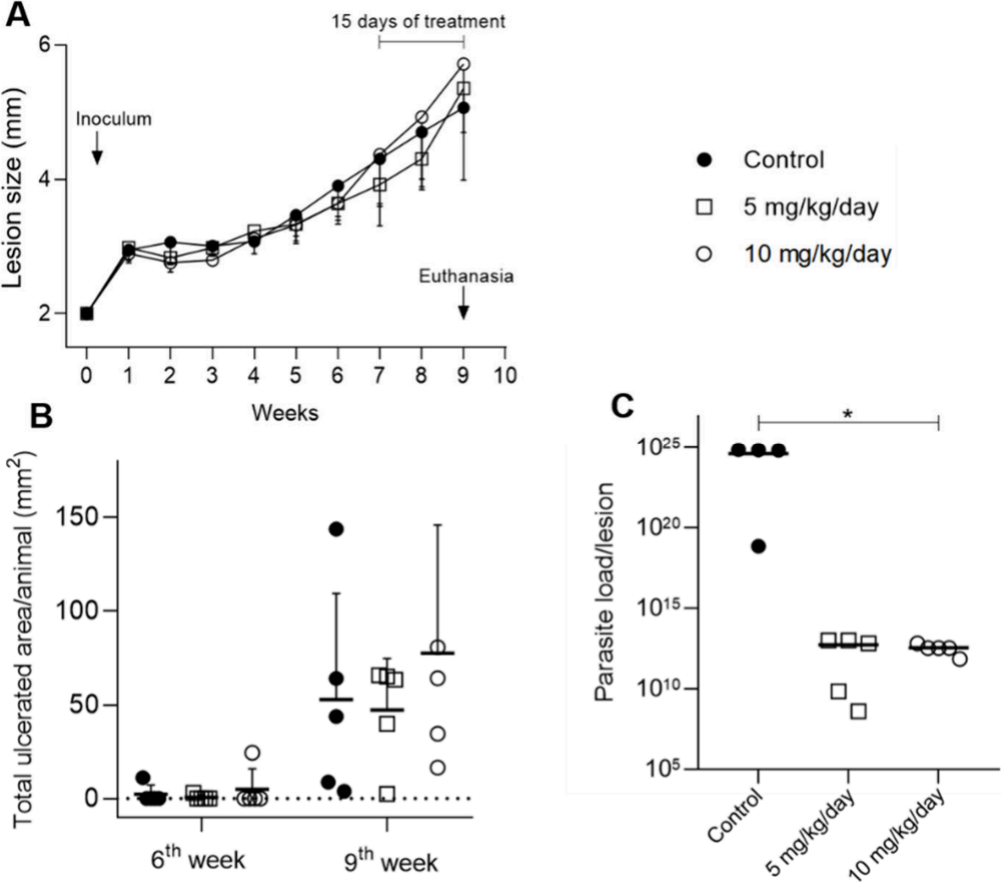
*In vivo* assay with intraperitoneal injection of beauvericin (**1**). BALB/c mice infected with 10^6^*L. amazonensis* metacyclic promastigotes were treated with **1** at 5 mg/kg/day (empty square), 10 mg/kg/day (empty circle) or solution vehicle only (black circle) for 15 days via IP. (A) Mean lesion obtained weekly. (B) Ulcerated area, obtained after the end of treatment. (C) Parasite load obtained by the limiting dilution assay. *p = 0.0157 comparing control group vs. beauvericin (**1**) treated groups.

Two independent *in vivo* assays were performed, comparing different routes of administration using female BALB/c mice infected with metacyclic *L. amazonensis* promastigotes at the base of the tail. Animals were treated with two distinct concentrations of **1** (5 and 10 mg/kg/day) intraperitoneally for 2 weeks after lesions were established (seventh week postinoculation) (Figure 4). Concerning the lesion evolution and ulcer development observed for untreated and treated animals (Figure 4A,B), mean sizes were similar during and post-treatment. Interestingly, after processing the lesion samples by limiting dilution assays, a significant decrease in the parasite burden was detected for both beauvericin (**1**) dose schemes (*p = 0.0157, Figure 4C).

Next, we evaluated the potential of beauvericin (**1**) topical application directly on the mice’s lesion caused by the parasite. Application of a higher dosage of **1** has been excluded as similar effects on parasite burden have been achieved in the previous assay (Figure 4). The treatment started after the establishment of lesions on the eighth week postinoculation, ending after 13 weeks postinoculation, with the euthanasia of the animals for sample processing. Lesions and weights were assessed weekly, covering the course of the infection.

**Figure 5:**
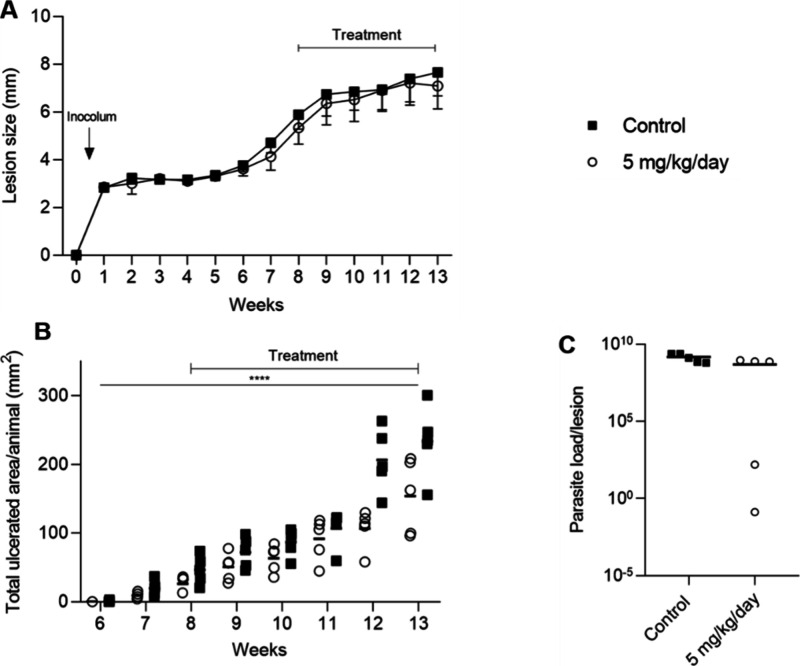
*In vivo* assay with topical treatment with beauvericin (**1**). BALB/c mice infected with 10^6^*L. amazonensis* metacyclic promastigotes were treated with 5 mg/kg/day (empty circle) or solution vehicle only (black square) for 32 days topically with 9.3 μL of solution using DMSO and glycerol (1:1) as the solution vehicle. All injections were given in 150 μL of DMSO and 0.9% aqueous NaCl solution (1:9). (A) Mean lesion obtained weekly. (B) Ulcerated area, obtained after the end of treatment. In (B), the *p* value was calculated for the area under the curve of lesion progression (AUC) between the control group and the group treated with 5 mg/kg/day. *p = <0.0001. (C) Parasite load obtained by the limiting dilution assay.

As in the intraperitoneal treatment (Figure 4), the topical treatment of **1** showed no difference in the mean lesions between the group treated with 5 mg/kg/day and the control group (Figure 5A). However, considering the total ulcerated area determined after the end of the treatment course, we observed a delay in the progression of the ulcers in mice treated with beauvericin (Figure 5B). Two animals treated with beauvericin (**1**) showed a decrease in the parasite load, obtained at the end of treatment by limiting dilution assays, compared to vehicle-treated animals (Figure 5C).

Several studies have reported the insecticidal potential of **1** against *Calliphora erythrocephala*, *Aedes aegypti*, and *Spodoptera frugiperda*, including recent studies investigating the role of **1** sources in microbiota-pathogen interactions.^18−20^ The role of *Beauveria bassiana* in fungal management has also been investigated, with its antifungal activity against *Botrytis cinerea* being reported, highlighting the presence of metabolites such as beauvericin (**1**) produced by *B. bassiana*.^21,22^

Beauvericin (**1**) is produced by several fungi, including *B. bassiana*, *Fusarium* ssp.,^17,23,24^ as well as *A. terreus*. Beauvericin has been described as a potent cytotoxic agent against tumor cell lines.^14,15^ Its activity against neoplastic cells (e.g., cervix carcinoma, adenocarcinoma, and metastatic colorectal adenocarcinoma) is expressed in the micromolar range. The migration inhibitory activity of **1** in two metastatic cancer cell lines has also been investigated using PC-3 M (prostate cancer cell) and MDA-MB-231 (breast cancer cell) lines.^25^ Similar results have been observed for **1** against human, animal, and plant pathogenic bacteria.^26−31^

Our results (Table 1) showed that **1** is active at low concentrations against both life cycle stages of *Leishmania* protozoan parasites that cause cutaneous leishmaniasis. In our morphological analysis of *L. amazonensis* promastigotes exposed to **1**, the frequency of typical promastigote forms decreased as concentrations of **1** increased, consistent with the viability impairment determined by the MTT assay. The EC_50_ of beauvericin (Table 1) aligned with the detection of the abnormal morphotypes presented in Figure 2. Beauvericin (**1**) also altered the promastigote morphology of *L. braziliensis*, but more markedly at the lowest concentrations tested (Figure 1). Observing live cultures before the fixation of thick smears revealed no motility at the three highest concentrations compared to the control group (data not shown), suggesting a leishmaniaostatic effect for **1**. Generally, cell bodies varied in shape but retained flagella. The “leaf-like” form had the highest frequency in the presence of 2.4 μM of **1** (>EC_50_), with the presence of flagella, a preserved nucleus, and kDNA, yet apparently not viable enough, given the EC_50_ obtained (Table 1, Figure 1).

Few organic and inorganic compounds have been tested for their effects on parasite morphology, revealing that rounding cell bodies generally indicates cell stress. At nanomolar concentrations, aryl-quinuclidine caused rounding promastigotes with plasma membrane fractures associated with the depletion of the 14-desmethyl endogenous sterol pool.^32^

Not only cell rounding but also mitochondrial changes, chromatin condensation, and nuclear and DNA fragmentation are features of a cell death process similar to apoptosis in *Leishmania*.^33^ Previous studies have detected morphological changes when *L. amazonensis* and *L. braziliensis* promastigotes were exposed to metallic compounds. Incubation of *L. amazonensis* promastigotes with copper II complexes for 36 h induced flagellar aberrations and cell body rounding.^34^ Cells presenting “round”, “aberrant”, and “sperm-like” aberrations were also found in *L. amazonensis* and *L. braziliensis* cultures exposed to gold-based compounds.^35,36^

Studies relying on the direct impact caused by antileishmanial candidates on promastigote morphology are scarce in the literature. Our results show, for the first time, that beauvericin (**1**) is capable of modifying the protozoan parasites in a drastic way, but with no suggestion of membrane permeability alteration. Beauvericin (**1**) increases ion permeability in biological membranes by forming essential cations complexes.^37^ In this sense, we evaluated the membrane permeability of promastigotes incubated for 24 h with **1** ∼EC_50_ (1.0 μM) by measuring ethidium bromide fluorescence, but no alteration was observed when compared with control cells (data not shown). The beauvericin (**1**) antiparasitic mechanism of action still deserves further investigation considering its effectiveness against *Leishmania* parasites.

Our *in vitro* assays demonstrated a potent reduction of intracellular amastigotes, with a slightly better activity when compared to axenic parasites (Table 1). The reduction in the infection rates and parasite burden reflected a significant decrease in the infectivity indexes for *L. amazonensis* and *L. braziliensis*. The absence of amastigotes in infections incubated with beauvericin (**1**) at 1.6 μM was very pronounced and empty macrophagic vacuoles indicate that **1** directly affected intracellular parasites, although host cells were well preserved (Figures 2 and 3). One study investigating *Aspergillus* secondary metabolites against *L. amazonensis* revealed that kojic acid led to a 79% inhibition of intracellular amastigotes with an EC_50_ (27.8 μg/mL), >250-fold higher than our results.^38^

In addition to macrophages having loss sensitivity to beauvericin (**1**), previous findings demonstrated that CHO-K1 cells are sensitive to **1** in equivalent periods of incubation used in our assays,^39^ while others showed that 50% culture keratinocytes were also affected by **1** at 3.9 μM.^15^ In our hands, L929 fibroblasts were more resistant to beauvericin (**1**) incubation for 24 h (45.5 μM). Based on these findings and added to the facts that amastigotes, clinical forms of relevance in leishmaniasis, are quite sensitive to **1** with EC_50_ = 0.5–0.9 μM (Table 1), we advanced to *in vivo* studies to evaluate the role of beauvericin (**1**) treatment in murine lesion evolution induced by *L. amazonensis*.

Beauvericin (**1**) activity has already been investigated in models of tumor induction in BALB/c mice with allografts, in which the intraperitoneal administration of **1** at 5 mg/kg/day results in the accumulation of **1** in tumors.^15^ Also, **1** significantly reduced weight loss, diarrhea, and mortality in mice with induced colitis, which mimics Crohn’s disease, also decreasing serum levels of TNF-alpha and IFN-gamma in a concentration-dependent manner. Therefore, beauvericin (**1**) can inhibit proliferation and activation, regulate cytokine profiles and induce apoptosis in activated T cells.^40^

Such results encouraged us to evaluate the effect of **1** on murine leishmaniasis at 5 and 10 mg/kg/day using the same mouse strain that is extremely susceptible to *L. amazonensis* infection. As observed in Figure 4, a significant reduction in lesion parasite load (*p* = 0.0157) for both beauvericin (**1**) treated groups was observed, despite clinical aspects remaining the same when compared to mock-treated mice (Figure 4B,C). However, when the topical application was conducted directly on the lesion site, i.e., topical route, a significant reduction of the total ulcerated area was observed in comparison to untreated animals (AUC; *p* < 0.0001) at the end of the treatment period (Figure 5B), leading us to assume that the action of **1** may depend on the chosen route of administration. Although different parasitological parameters were evaluated and showed different responses, the potential healing action of beauvericin, even if partial, was observed in an infection model of high susceptibility to the host (BALB/c mouse-PH8 *L. amazonensis* strain), suggesting that there may be a promising activity of beauvericin (**1**) in studies in which its administration can be improved.

Besides the fact that beauvericin (**1**) exhibits strong efficacy against *Leishmania* parasites, our findings are the first to report the application of a topical solution of **1** on skin lesions induced by a protozoan, opening perspectives for advancing the design of new assays with different dosing schemes and perspectives regarding drug delivery systems of a leishmanicidal candidate. We showed promising *in vitro* and *in vivo* leishmanicidal effects of beauvericin (**1**), emphasizing that systemic inoculation of **1** led to a decrease in the lesion site parasite load, whereas its topical administration delayed the progression of ulcers, a cure criterion established for CL management that should be exploited in novel experimental schemes of drug tests. The potential of beauvericin (**1**) as a leishmanicidal agent highlights the importance of exploring natural products in the development of antiparasitic treatments.

## Experimental Section

### General Experimental Procedures

The optical rotation was measured on a Jasco P-2000 polarimeter. NMR spectra were obtained at 25 °C, with their own solvent as an internal standard, by using a Bruker AV-600 spectrometer operating at either 600 MHz (^1^H) or 150 MHz (^13^C) with a 2.5 mm cryoprobe. HPLC-PDA-MS analyses were carried out on a Waters chromatography system consisting of a Waters 2695 Alliance control system coupled to a Waters 2696 UV–visible spectrophotometric detector with photodiode array detector, connected sequentially to a Waters Micromass ZQ 2000 mass spectrometry detector operated using Empower platform. Analyses were performed using a Waters C_18_ X-Terra reversed-phase column (4.6 × 250 mm, 5 μm). The mass spectrometer detector was optimized using the following conditions: capillary voltage, 3 kV; temperature of the source, 100 °C; desolvation temperature, 350 °C; ESI mode, acquisition range, 150–1200 Da; gas flow without cone,: 50 L h ^–1^; desolvation gas flow, 350 L h ^–1^. Samples were diluted in MeOH at a concentration of 2 mg mL^–1^. UPLC-QToF-HRMS analyses were performed on a Waters Acquity H-Class UPLC instrument coupled to a Xevo G2-XS Q-TOF instrument with an electrospray ionization (ESI) interface. The chromatographic separation was performed using a Waters Acquity UPLC BEH column (RP18, 2.1 × 100 mm, 1.7 μm) with a mobile phase composed of H_2_O + 0.1% formic acid (A) and MeCN + 0.1% formic acid (B). The following gradient was applied at a flow rate of 0.5 mL/min: 10% to 50% B in 6 min, from 50 to 98% until 9 min, then setting it back to 10% B at 9.10 min, and keeping it at 10% B until *t* = 10 min. The HRESIMS data were acquired in positive ion mode.

### Plant Material

Authenticated *Axonopus leptostachyus* was collected in Poconé, Pantanal of Mato Grosso, Brazil, in April 2012. A voucher specimen is preserved at the Herbarium of the Federal University of Mato Grosso, Brazil (Voucher no 40.492).^41,42^

### Fungus Isolation and Identification

The endophytic fungus *Aspergillus terreus* P63 was isolated from the roots of *Axonopus leptostachyus* after surface sterilization.^41,43^ The pure fungal strain was obtained through serial transfers onto PDA and was maintained as *Aspergillus terreus* P63 in the Laboratory of Biotechnology and Microbial Ecology, Brazil. The pure *A. terreus* P63 strain was submitted to molecular identification by sequencing the ITS regions using the primers ITS1 and ITS4.^4444^ The nucleotide sequence was deposited in the GenBank database with the accession number KJ439155.^42^

### Cultivation and Isolation of Metabolites of *A. terreus* P63

The endophytic fungus strain *A. terreus* P63 was grown in 27 500 mL Schott flasks, each filled with 30 mL of distilled water and 25.0 g of rolled oats. The media were autoclaved twice on consecutive days at 121 °C for 20 min each time. After sterilization, four small pieces (2 × 2 cm) of PDA medium, containing biomass of the isolated *A. terreus* P63, were added to each flask. The inoculated media were incubated in stationary at 25 °C in the dark for 30 days. After the incubation period, the cultures were combined, blended in MeOH (100 mL) and extracted for 5 h. After filtration, the MeOH extract was defatted by liquid–liquid partitioning with hexane (3 × 500 mL). Then, the MeOH fraction was evaporated to dryness, solubilized in H_2_O/EtOAc 3:5 (v/v) and subjected to a liquid–liquid partitioning between H_2_O and EtOAc. The EtOAc fraction (named AcRP) was evaporated to dryness to yield 10.142 g, which was subjected to bioassay-guided fractionation.

The active AcRP organic fraction (10.142 g) was subjected to an open dry column chromatography of C_18_-derivatized silica-gel eluted with 1 L of each eluent: 100% H_2_O, 9:1 H_2_O/MeOH; 8:2 H_2_O/MeOH, 7:3 H_2_O/MeOH, 6:4 H_2_O/MeOH, 1:1 H_2_O/MeOH, 4:6 H_2_O/MeOH, 3:7 H_2_O/MeOH, 2:8 H_2_O/MeOH, 1:9 H_2_O/MeOH and 100% MeOH, to afford 12 major subfractions, AcRP-1 to AcRP-12. After evaporation, the subfractions obtained were analyzed by thin layer chromatography (TLC) using anisaldehyde/sulfuric acid reagent, followed by heating at 100 °C for 5 min. Fluorescent spots were visualized under UV light at λ_max_ values of 254 and 366 nm.

The active fraction AcRP-10 was separated by size-exclusion chromatography on a Sephadex LH-20 column and eluted with MeOH resulting in 399 fractions of 10 mL each. Fractions were combined in seven additional fractions (AcRP-10-A to AcRP-10-G) after analysis by thin-layer chromatography (TLC) under the same conditions described above. Fraction AcRP-10-C was identified as containing beauvericin (**1**) (315.0 mg).

Fraction AcRP-12 was separated by size-exclusion chromatography on a Sephadex LH-20 column, eluted with MeOH to yield 300 fractions of 10 mL each. Fractions were combined in eight (AcRP-12-A to AcRP-12-H) additional fractions after analysis by TLC (see above). The active fraction AcRP-12-D was identified as containing compound **1** (619.0 mg). The purity of **1** was measured as >98% by ^1^H NMR analysis, by calculating the average percentage of impurities peak areas related to beauvericin peak areas by integration of ^1^H NMR signals.

### Parasites and Mammalian Cell Cultivation

Promastigotes of *Leishmania* (*Leishmania*) *amazonensis* (IFLA/BR/67/PH8) and *L*. (*Viannia*) *braziliensis* (MHOM/BR/91/H3227) were grown in M199 medium pH 7.4 (Sigma-Aldrich) supplemented with 40 mM HEPES (pH 7.4), 0.1 mM adenine, 0.0001% biotin, 0.0005% hemin, 10% fetal bovine serum (FBS) and 5 μL/mL penicillin/streptomycin (10 mg/mL) at 25 °C. *L. braziliensis* was grown in the medium described above supplemented with 5% sterile human male urine and 10% FBS.

Epimastigotes of *Trypanosoma cruzi* (Y strain) were grown in LIT medium^44^ supplemented with 10% FBS and 5 μL/mL penicillin/streptomycin (10 mg/mL) at 28 °C.

Fibroblasts (L929 strain) were cultured in RPMI 1640 medium supplemented with 40 mM HEPES (pH 7.4), 0.1 M sodium pyruvate, 200 mM l-glutamine, 10% FBS and 5 μL/mL penicillin/streptomycin (10 mg/mL) at 37 °C under 5% CO_2_ atmosphere.

To obtain bone marrow-derived macrophages (BMDM), precursor cells were extracted from the femurs and tibias of female BALB/c mice for differentiation. The medullary cavities of the bones were washed with a 5 mL syringe and a 21G needle with Roswell Park Memorial Institute (RPMI 1640) medium (Gibco-Invitrogen) supplemented with 20% FBS and 30% L929 fibroblast culture supernatant in 75 mm Petri dishes. Recovered cells were kept at 37 °C with a 5% CO_2_ atmosphere. After 7 days, differentiated BMDM samples were collected with fresh RPMI medium after scraping the plate with a sterile cell scraper (Biofil). The Ethics Committee on Animal Use of the University of Campinas (CEUA-UNICAMP) (protocol 6384-1/2024) approved all mice using procedures.

### Cell Viability Assays

*L. amazonensis* and *L. braziliensis* promastigotes and *T. cruzi* epimastigotes (5 × 10^6^/well), L929 fibroblasts (5 × 10^4^/well), and BDMD (4 × 10^5^/well) were incubated with increasing concentrations of beauvericin (**1**) (solubilized in sterile pure DMSO) in 96-well plates. After 24 h incubation, 30 μL of MTT (3-[4,5-dimethylthiazol-2-yl]-2,5-diphenyltetrazolium bromide; Sigma-Aldrich) (5 mg/mL) was added to each well and the plates were incubated for 3 h for subsequent reaction interruption by the addition of 30 μL of 20% SDS. Absorbance of formazan, the product of MTT reduction, was determined in a Spectrophotometer BioTek Synergy HT microplate reader with reference and test wavelengths of 650 and 600 nm, respectively.^45^ The morphology of parasites was determined as previously described.^34^ Briefly, 10 μL culture aliquots from control and (**1**)-incubated promastigotes were dispensed on glass slides for the preparation of thick smears. The smears were dried at room temperature for 24 h, fixed with pure MeOH for 1 min and stained with an InstantProv kit (NewProv). At least 200 promastigotes were observed for each concentration of **1** (0, 0.6, 1.2, 2.4, 4.8, and 9.6 μM) by optical microscopy (Nikon Eclipse E200). *Leishmania* promastigotes were quantified in percentages of the total cells counted according to the following morphotypes: “typical”, “aberrant”, “slender”, “leaf-like”, “sperm-like”, and “debris”.

### *In Vitro* Infection Assays

Four ×10^5^ BMDM were seeded on 13 mm glass coverslips in 24-well plates for evernight for adherence and infected with stationary phase promastigotes of *L. amazonensis* (multiplicity of infection (MOI) = 5) or *L. braziliensis* (MOI = 10) for 24 h at 34 °C with 5% CO_2_ atmosphere in triplicate for each condition described below. Cells were washed with warm PBS 1× to eliminate noninternalized promastigotes, and beauvericin (**1**) was added at 0.4, 0.8, and 1.6 μM to a fresh medium of RPMI 1640. Infections were incubated for continuous 24 h and 48 h or 24 h plus 24 h with **1** and fresh medium addition (48 h-fm). At the end of each incubation period, coverslips were washed with warm PBS 1× and cell monolayers were fixed with pure MeOH for subsequent staining with Instant Prov Kit (NewProv). The coverslips were examined using the Invitrogen EVOS XL Core Cell Imaging System (Thermo Scientific) to determine infection rates (infected macrophages), intracellular parasite burden (mean of the amastigote number per 100 macrophages), and infectivity index (infection rates × intracellular parasite burden) by counting at least 300 host cells per condition.

### *In Vivo* Assays

Isogenic 35-day old BALB/c strain mice were obtained from the Central Animal Facility of CEMIB-UNICAMP and kept in Alesco (ALBR Indústria e Comércio LTDA) cages at 24 °C, photoperiod 12 h/12 h, and humidity of 55 g/m^3^ with water and food *ad libitum* at the Parasitology Animal Experimentation Laboratory, Institute of Biology - UNICAMP. Approximately 1 × 10^6^ stationary-phase promastigotes of *L. amazonensis* were inoculated at the mice tail base to establish localized lesions for subsequent rounds of treatment with beauvericin (**1**). Beauvericin was solubilized in sterile pure DMSO + aqueous NaCl 0.9% (1:9, v/v) and stored at 4 °C for daily administration of 5 and 10 mg/kg/day for 15 days (5 animals per group). The control group (*n* = 5 animals) received the same volume of the diluents. The beauvericin (**1**) solution (150 μL) was inoculated in each animal by the ip route daily for 15 days. Treatment started at the seventh week postinoculation, after the establishment of lesions in the infected animals, and ended at ninth week postinoculation.

The topical administration scheme was performed using **1** solubilized in sterile pure DMSO + glycerol (1:1, v/v) stored at 4 °C. Ten animals were used in this case, subdivided as (i) control group, receiving topical treatment with DMSO + glycerol (1:1) and (ii) group treated with 5 mg/kg/day. Beauvericin (**1**) was administered daily for 32 days by pipetting 9.3 μL of (**1**) solution (5 mg/kg/day) directly onto the lesion. The treatment was initiated at the eighth week postinoculation, after the establishment of the lesions in infected animals, and ended at the 13th postinoculation. Lesion diameters were assessed weekly with a digital caliper (Mitutoyo).

### Limiting Dilution

At the end of the treatment, animals were euthanized after anesthesia (240 mg/kg ketamine hydrochloride at 50 mg/mL and 30 mg/kg xylazine hydrochloride 2%), followed by cervical dislocation according to the recommendations of the National Council for the Control of Animal Experimentation (CONCEA) and approved by the Ethics Committee on Animal Use (CEUA - UNICAMP - Protocol #6384–1/2024).

Tail lesions were scraped using a scalpel and mechanically macerated in 1 mL of sterile 1× PBS using an autoclavable plastic homogenizer. After tissue maceration, the contents were centrifuged at 400*g* for 10 min at 4 °C. The supernatant was centrifuged at 1,500*g* for 8 min at 4 °C to recover the parasites, and three washes with 1 mL of PBS 1× (pH 7.4) were performed. Subsequently and under the same centrifugation conditions, parasites were washed in 1 mL of acid medium 199 (M199, pH 4.8) followed by a wash in 1 mL of PBS 1× (pH 7.4). The resulting pellet was resuspended in 1 mL of M199 (pH 7.4), and 10 μL of this total content was added to the first well of 96-well plates, containing 150 μL of M199 (pH 7.4), to perform dilution with a multichannel pipet up to the 24th well of the plate. After 7 days, the plates were examined under a Leica DMi1 inverted microscope to identify and quantify the parasites present in the wells.^46^

### Data Analysis

Independent *in vitro* infection and *in vivo* assays were conducted by comparing the untreated control group with the treated group, and the results shown are the means ± S.D., analyzed by Student’s *t* test and ANOVA using *GraphPad Prism8* software.

## Supporting Information Available

Isolation scheme of **1**, ^1^H, ^13^C, 2D NMR and MS spectra of beauvericin, images of the fungus, (PDF)

## Dedication

Dedicated to Dr. Sheo B. Singh, retired from Merck, now with Drew University and Stevens Institute of Technology, for his pioneering work on bioactive natural products.
