# Supplementary material for: In Vitro and In Vivo Leishmanicidal Activity of Beauvericin
Source: J Nat Prod. 2024 Dec 3;87(12):2829–38. doi: 10.1021/acs.jnatprod.4c01098 (PMC11686502; doi:10.1021/acs.jnatprod.4c01098)
Supplement: Supplementary file 1 — np4c01098_si_001.pdf [file np4c01098_si_001.pdf]

Supplementary Information

For

In vitro and *In vivo* Leishmanicidal Activity of Beauvericin

Virlânio A. de Oliveira Filho,[†] Juliana R. Gubiani,[‡] Vitória D. Borgonovi,[‡] Felipe Hilário,[‡] Marcelo R. de Amorim,*[‡] Karen Minori,[†] Vitor K. da S. Bertolini,[†] Antonio G. Ferreira,[⊥] Helder L. Teles,[§] Fernanda R. Gadelha,[†] Roberto G. S. Berlinck,*[‡] and Danilo C. Miguel*[†]

[†]Instituto de Biologia, Universidade Estadual de Campinas, Campinas, 13083-862, SP, Brazil.

[‡]Instituto de Química de São Carlos, Universidade de São Paulo, CP 780, 13560-970, São Carlos, SP, Brazil.

[⊥]Departamento de Química, Universidade Federal de São Carlos, São Carlos, 13565-905, SP, Brazil.

[§]Instituto de Ciências Exatas e Naturais, Departamento de Biologia, Universidade Federal de Mato Grosso, Campus de Rondonópolis, 78735-901, Rondonópolis, MT, Brazil.

List of Supplementary Figures

Figure S1. Flowchart of preparation and fractionation of the extract of the fungus *A. terreus* P63 media to obtain the beauvericin (**1**).. S3
Figure S2. ^{1}H NMR spectrum of beauvericin (**1**) (600 MHz, DMSO-d_6)...................... S4
Figure S3. Expansion of the ^{1}H NMR spectrum of beauvericin (**1**) (600 MHz, DMSO-d_6). S5
Figure S4. Expansion of the ^{1}H NMR spectrum of beauvericin (**1**) (600 MHz, DMSO-d_6). S6
Figure S5. ^{13}C NMR spectrum of beauvericin (**1**) (150 MHz, DMSO-d_6)..................... S7
Figure S6. HSQC spectrum of beauvericin (**1**) (DMSO-d_6)... S8
Figure S7. HMBC spectrum of beauvericin (**1**) (DMSO-d_6). S9
Figure S8. COSY spectrum of beauvericin (**1**) (DMSO-d_6)... S10
Figure S9. Chromatogram of beauvericin (**1**) by HPLC-DAD. Monitored at 254 nm. S11
Figure S10. LR-ESI-MS spectrum of beauvericin (**1**) in positive ionization mode by HPLC-MS.. S12
Figure S11. HR-ESI-MS spectrum of beauvericin (**1**) in positive ionization mode by UPLC-QToF-HRMS. S13
Figure S12. Expansion of the HR-ESI-MS spectrum of beauvericin (**1**) in positive ionization mode by UPLC-QToF-HRMS... S14
Figure S13. Image of the fungus *Aspergillus terreus* P63 preserved in plate with potato dextrose agar.. S15

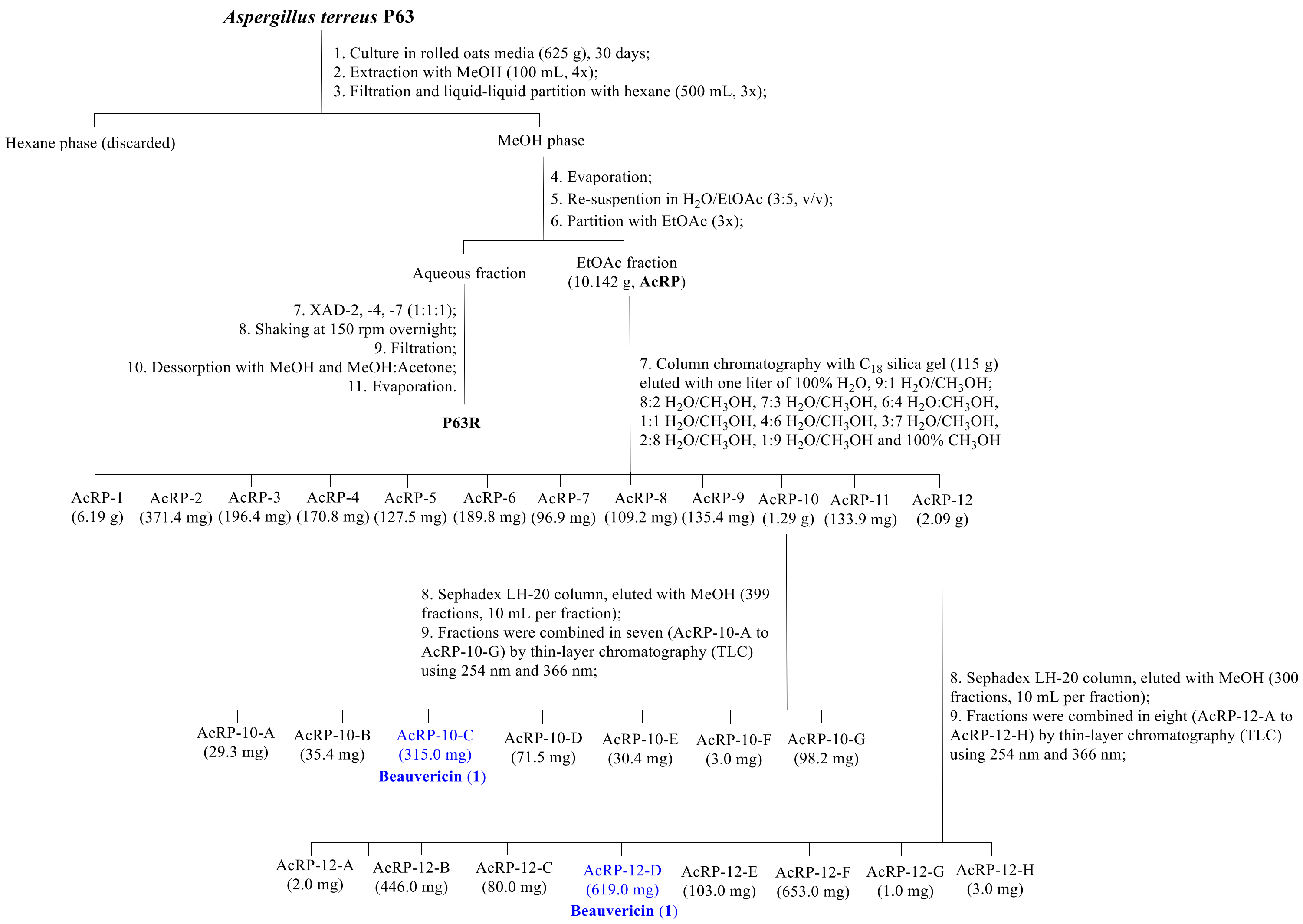

Figure S1. Flowchart of preparation and fractionation of the extract of the fungus *A. terreus* P63 media to obtain the beauvericin (**1**).

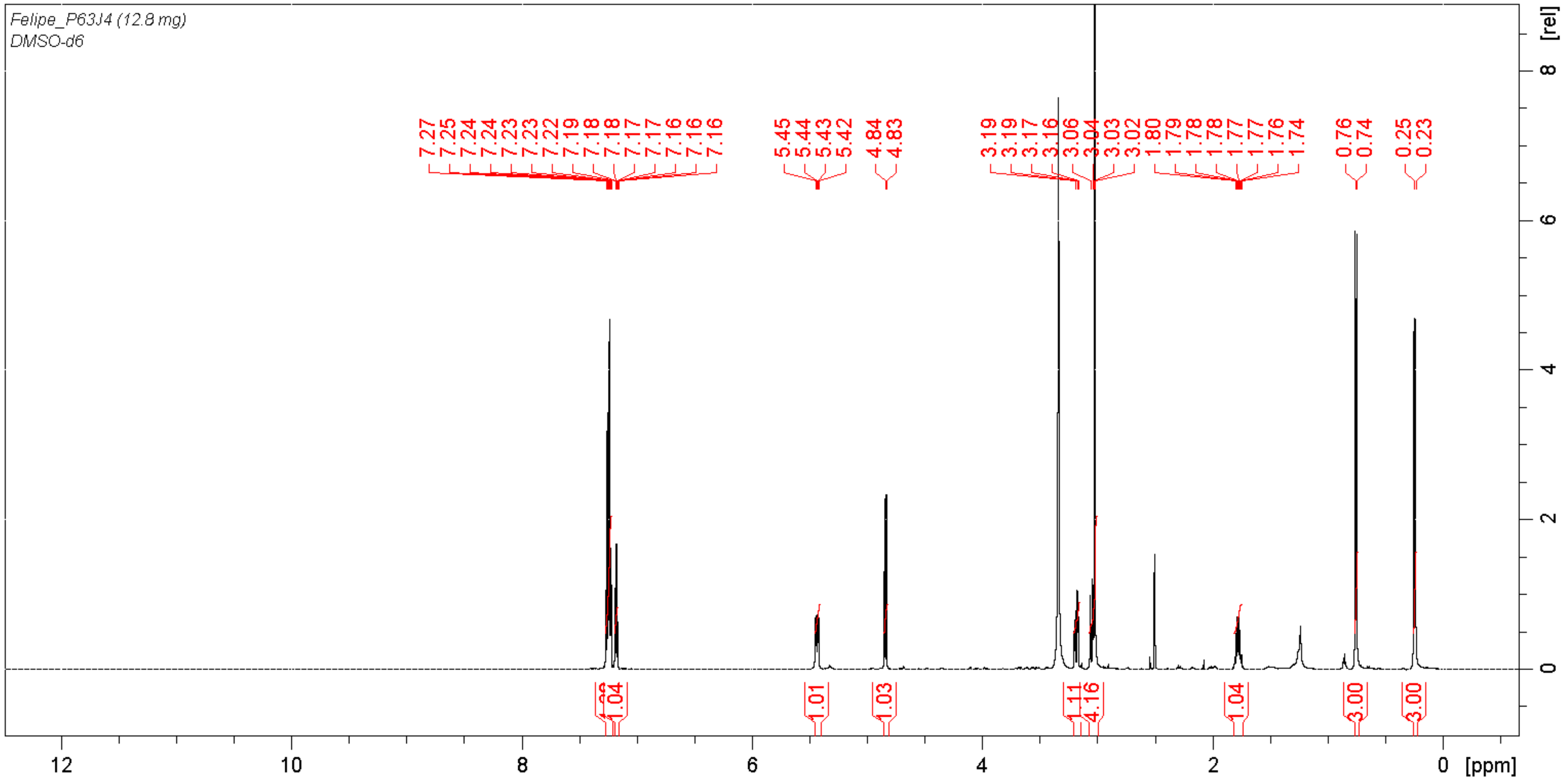

Figure S2. ^{1}H NMR spectrum of beauvericin (**1**) (600 MHz, DMSO-d_6).

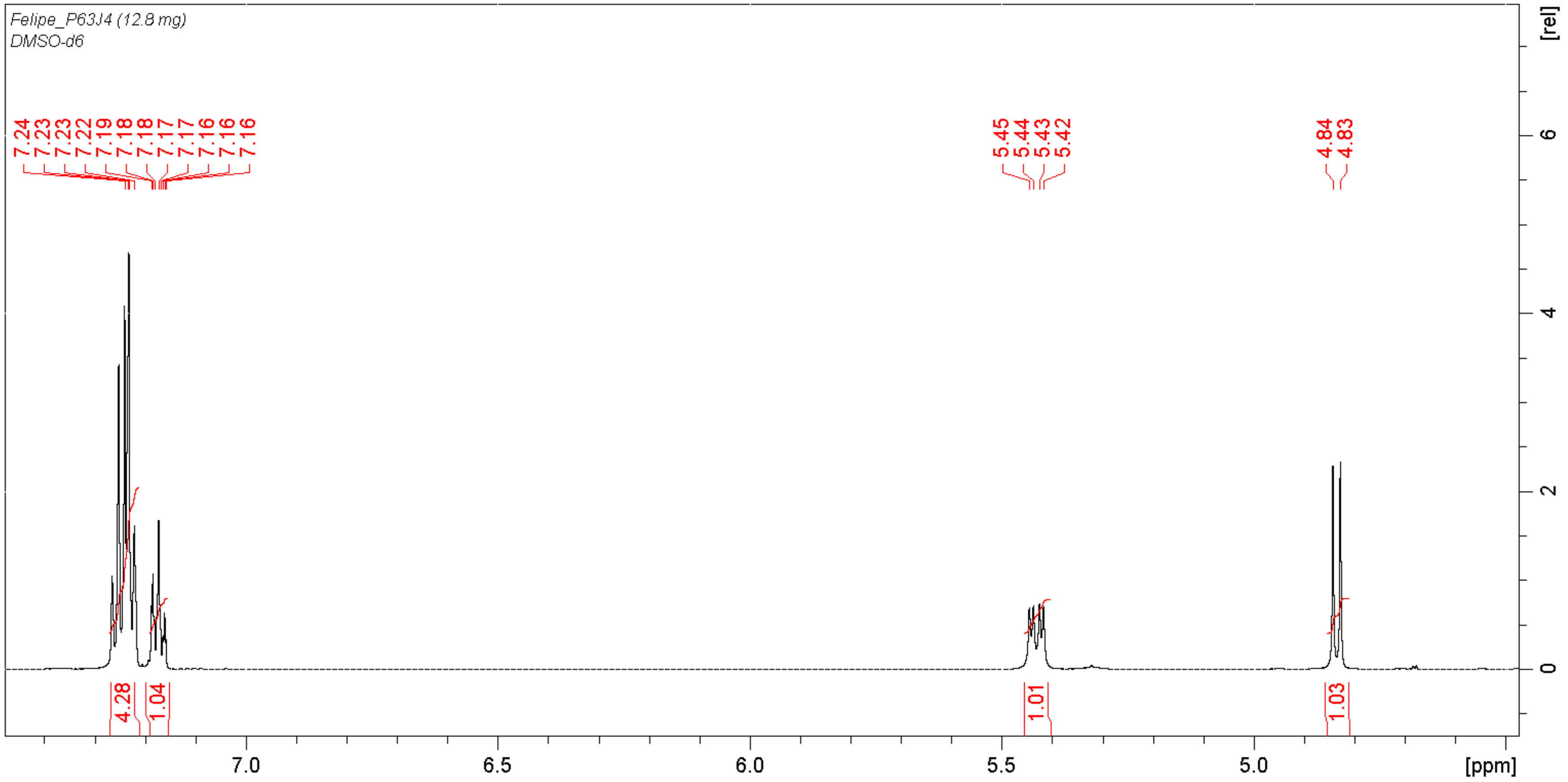

Figure S3. Expansion of the ^{1}H NMR spectrum of beauvericin (**1**) (600 MHz, DMSO-d_6).

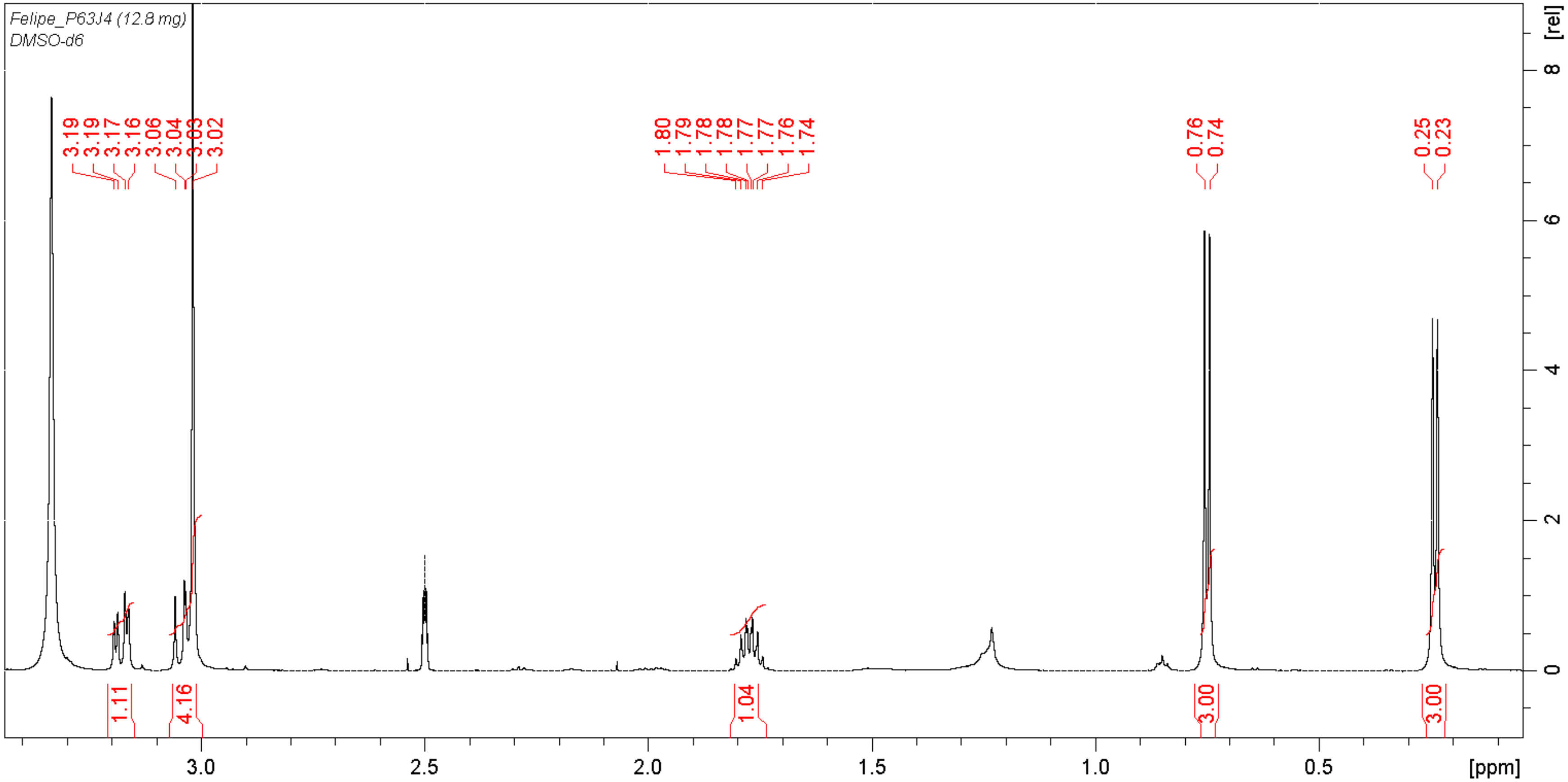

Figure S4. Expansion of the ^{1}H NMR spectrum of beauvericin (**1**) (600 MHz, DMSO-d_6).

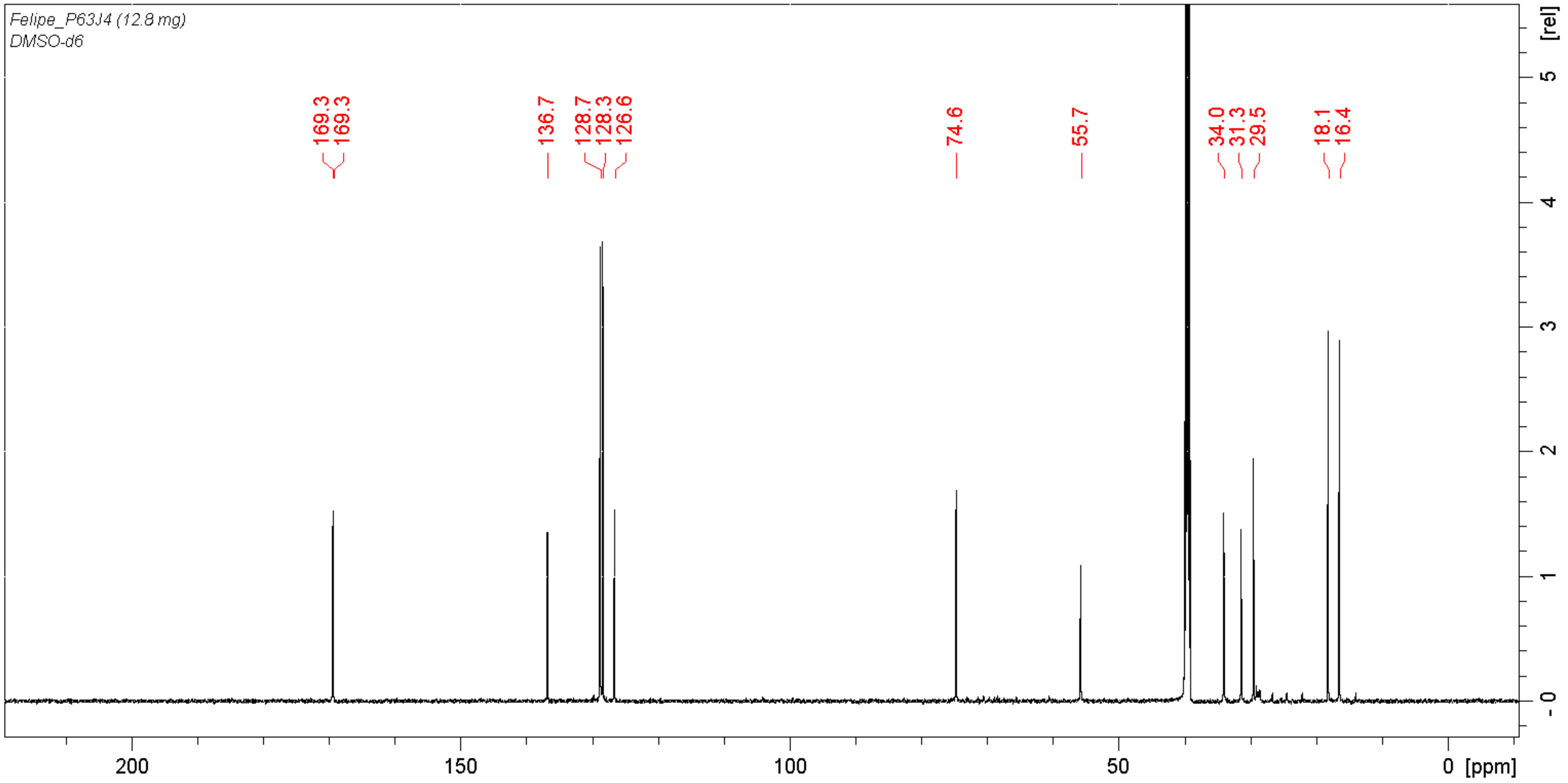

Figure S5. ^{13}C NMR spectrum of beauvericin (**1**) (150 MHz, DMSO-d_6).

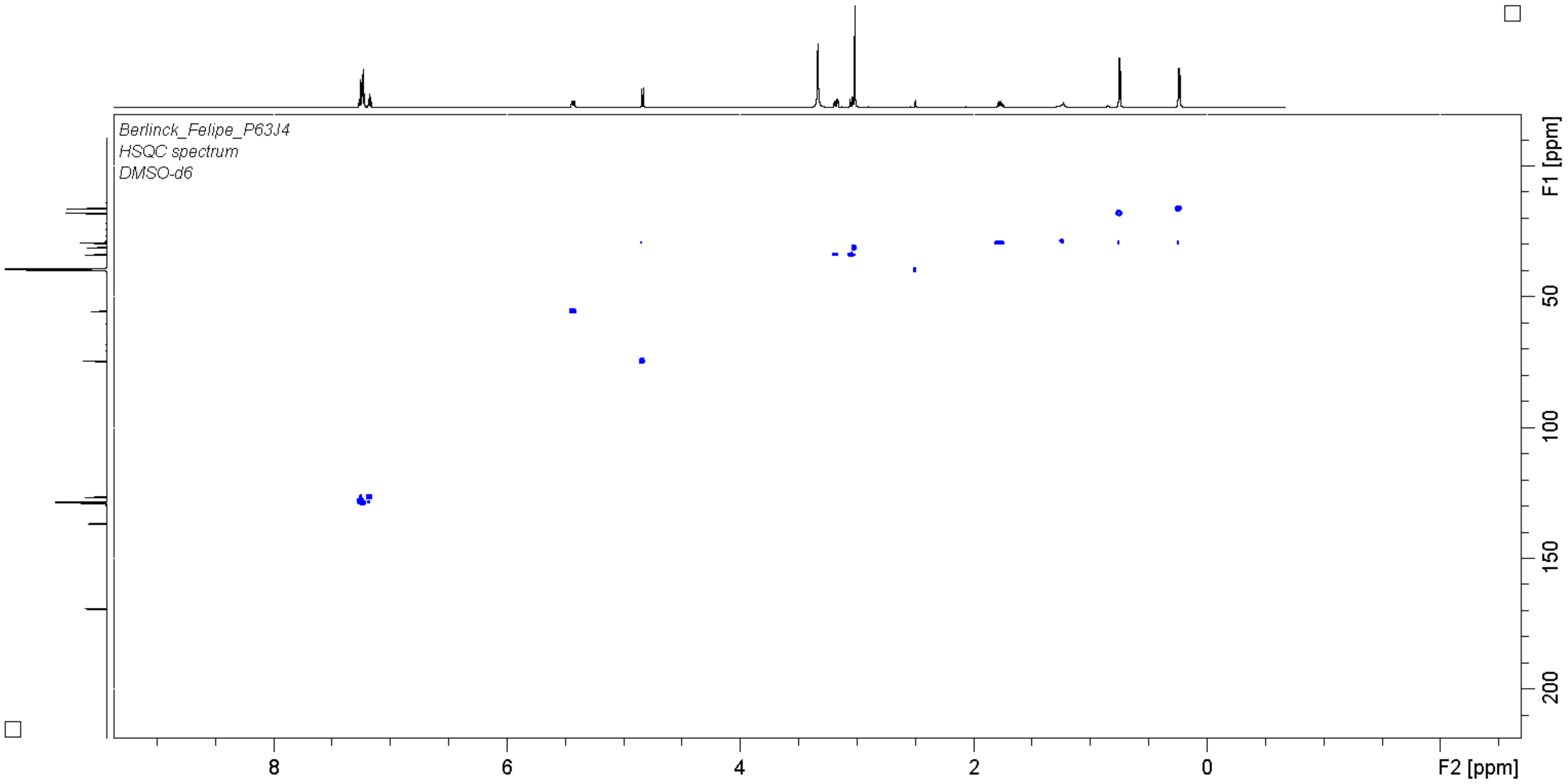

Figure S6. HSQC spectrum of beauvericin (**1**) (DMSO-d_6).

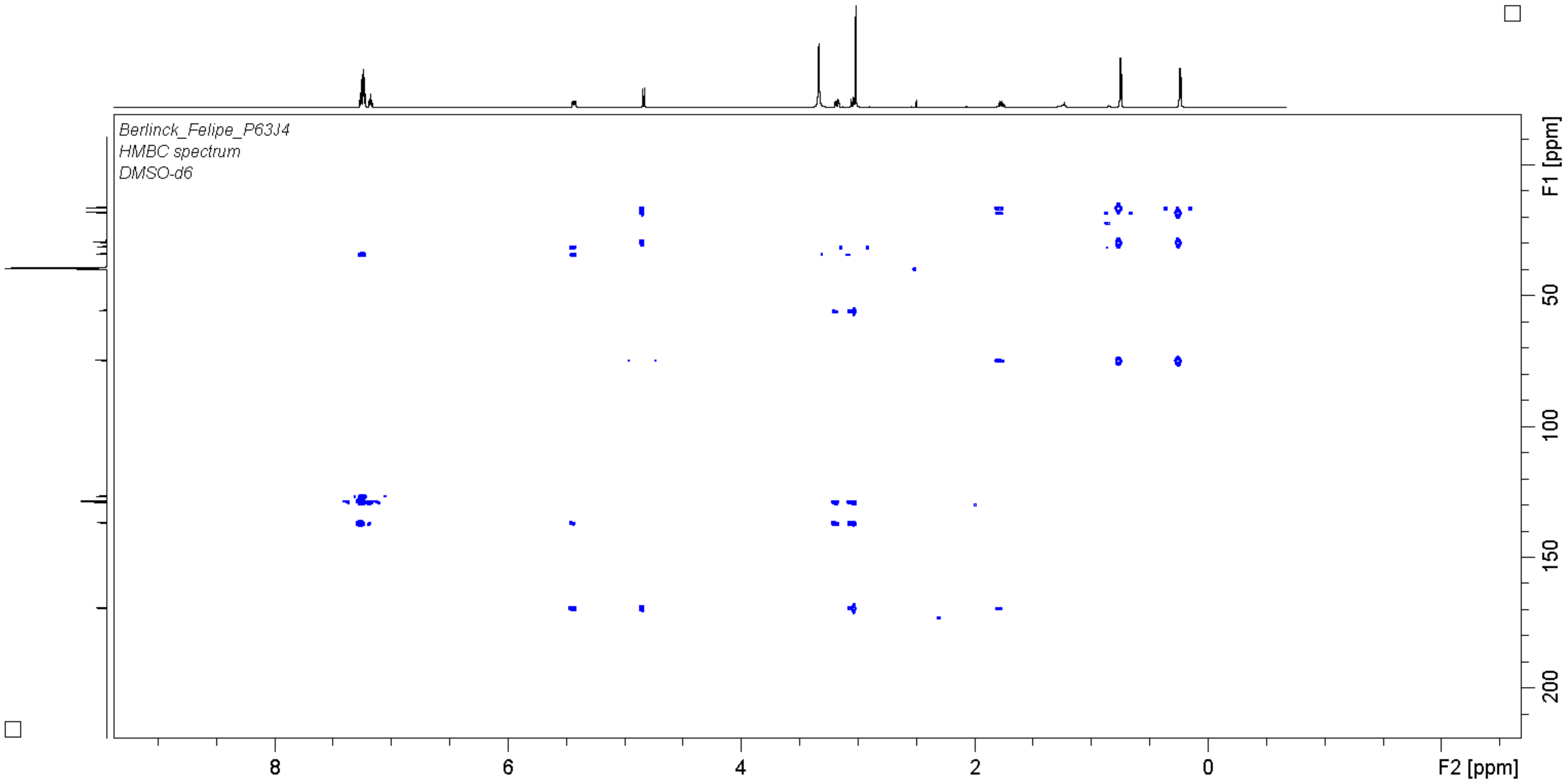

Figure S7. HMBC spectrum of beauvericin (**1**) (DMSO-*d*$_6$).

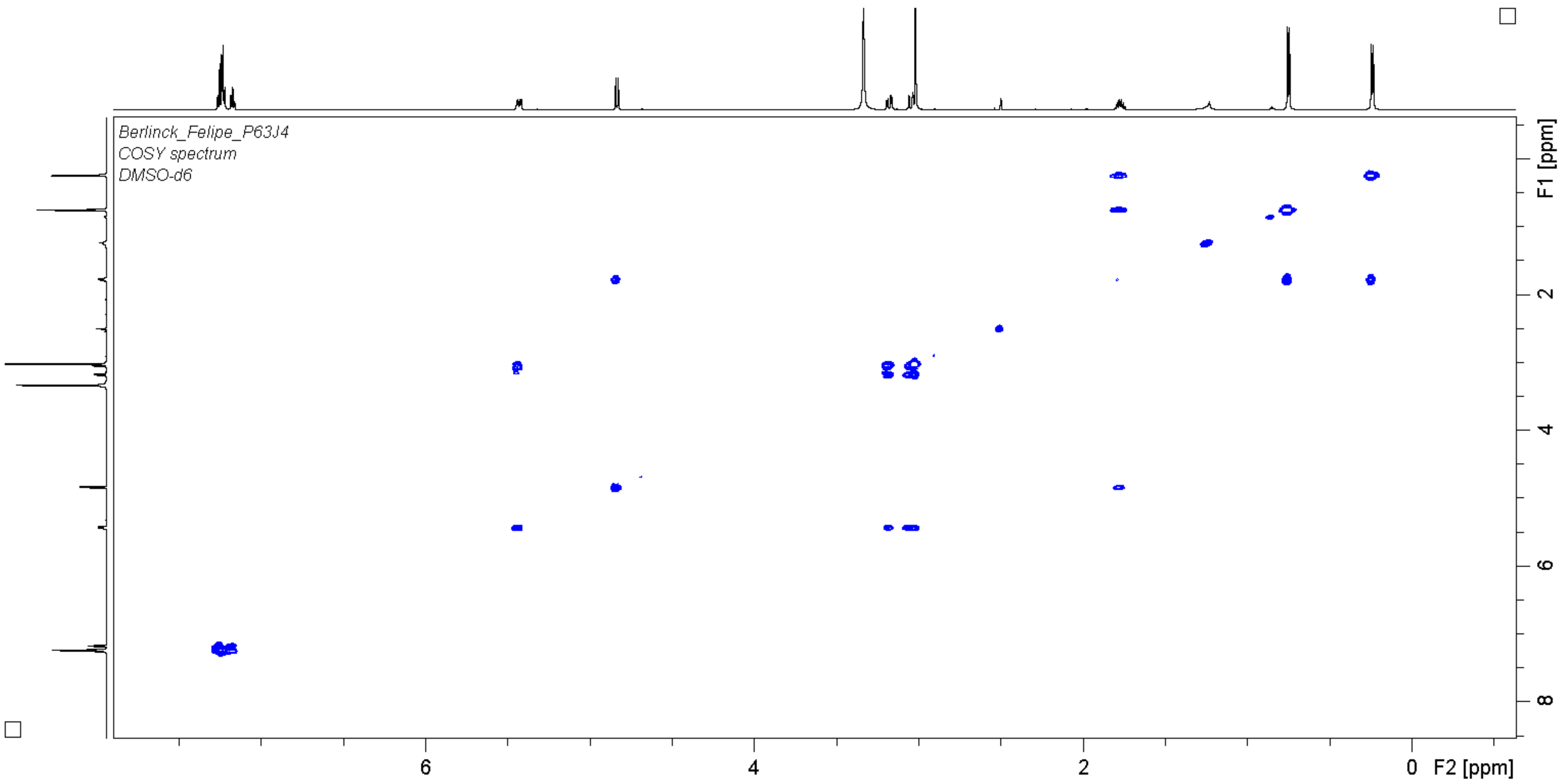

Figure S8. COSY spectrum of beauvericin (**1**) (DMSO-d_6).

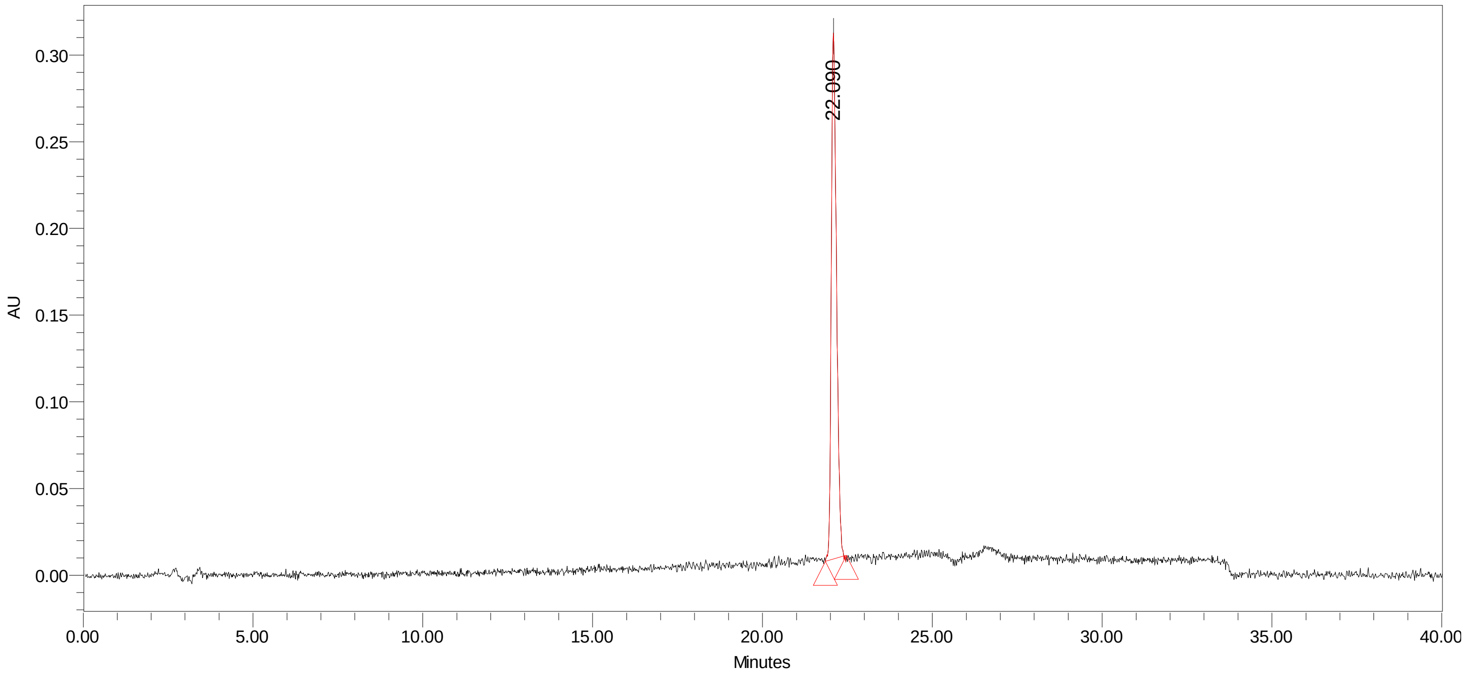

Figure S9. Chromatogram of beauvericin (**1**) by HPLC-DAD. Monitored at 254 nm.

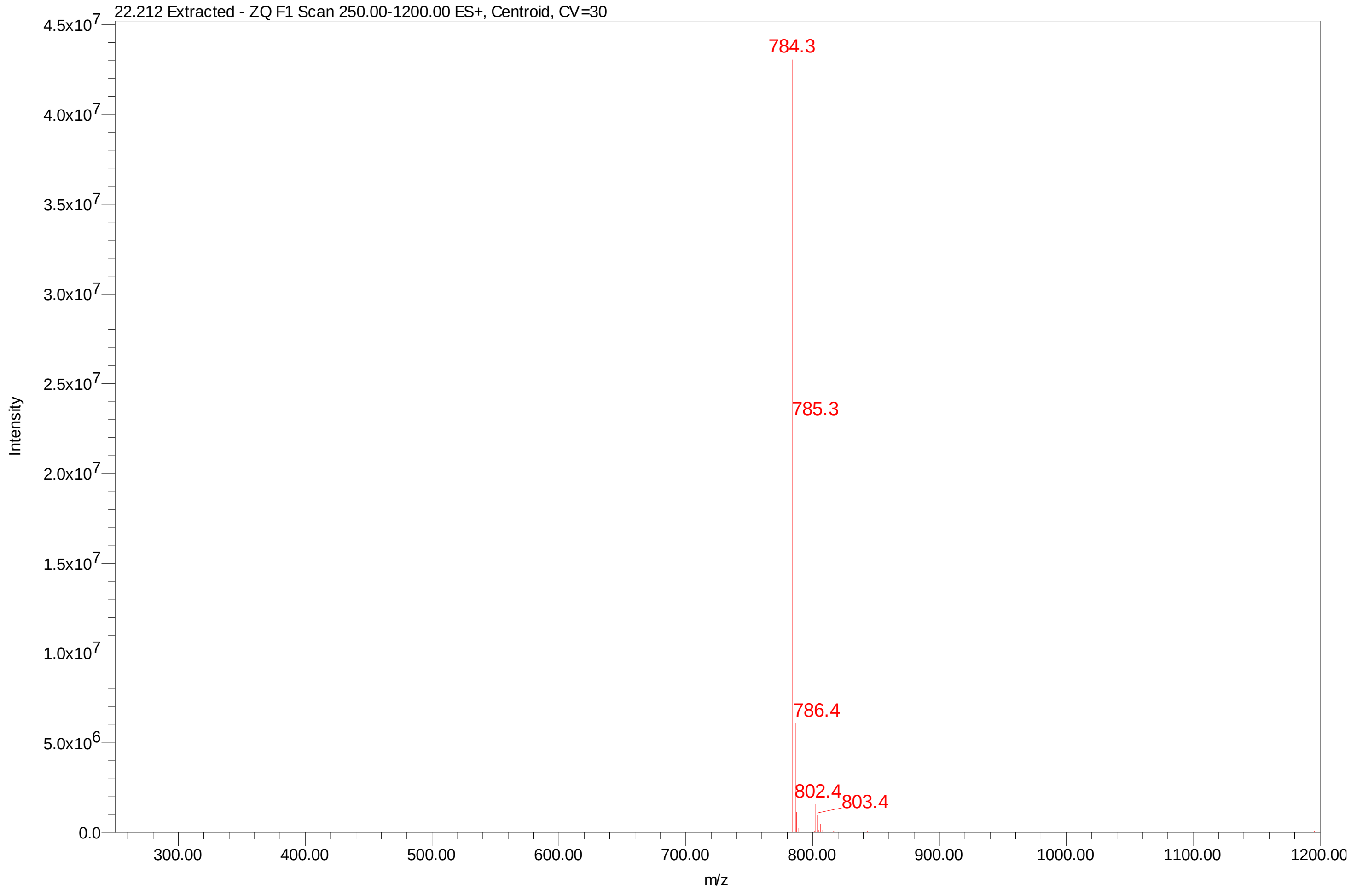

Figure S10. LR-ESI-MS spectrum of beauvericin (**1**) in positive ionization mode by HPLC-MS.

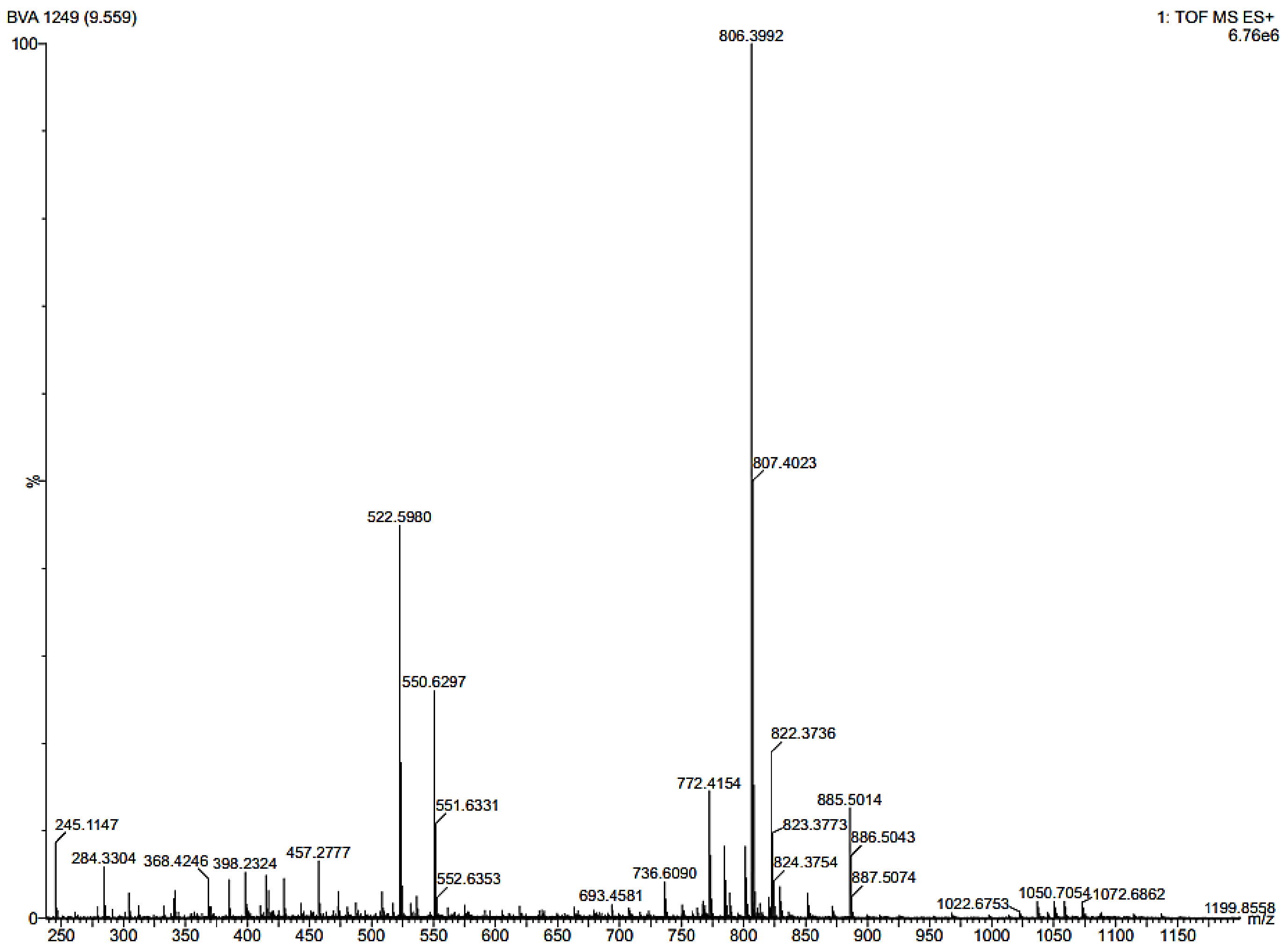

Figure S11. HR-ESI-MS spectrum of beauvericin (**1**) in positive ionization mode by UPLC-QToF-HRMS.

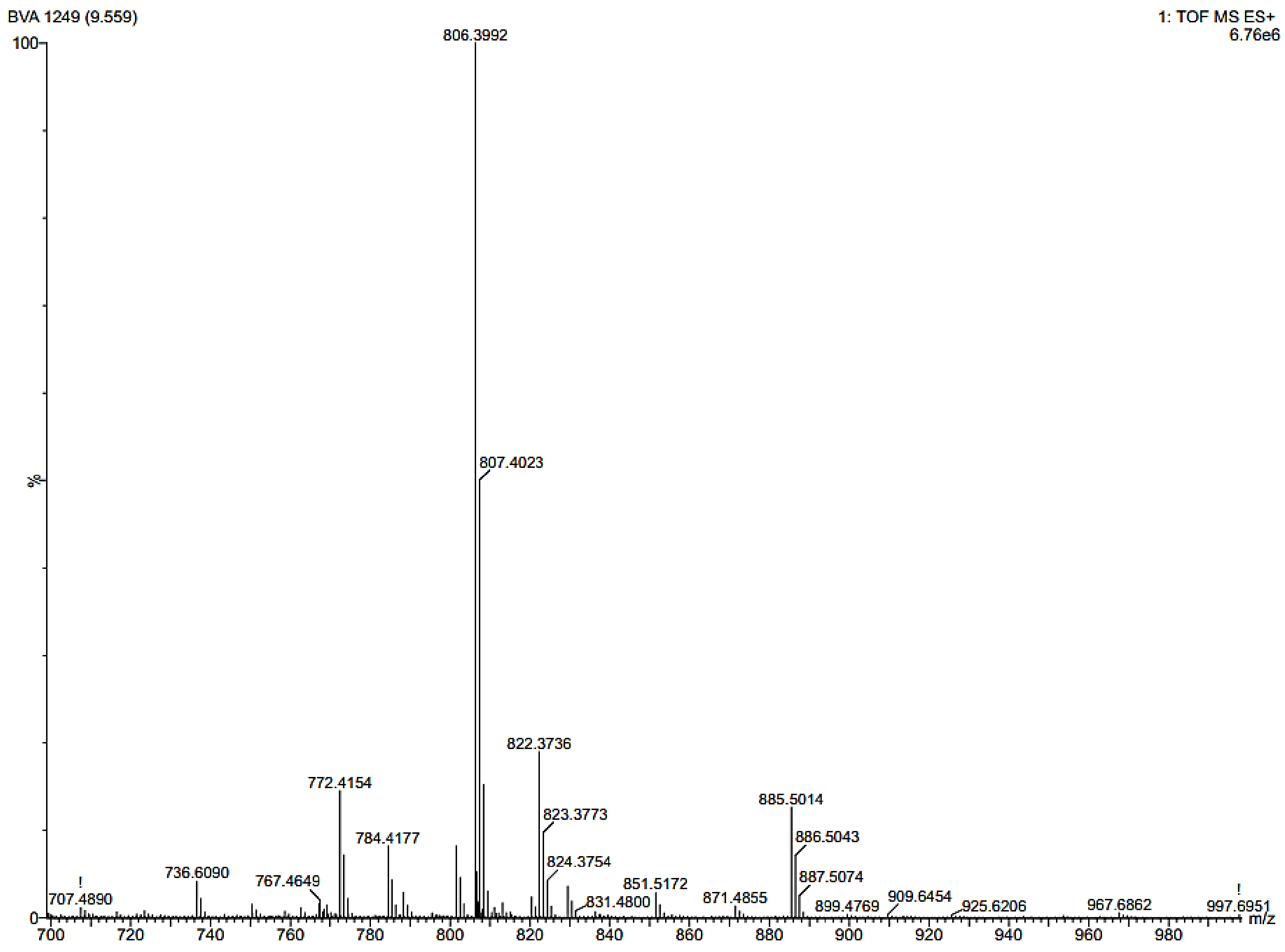

Figure S12. Expansion of the HR-ESI-MS spectrum of beauvericin (**1**) in positive ionization mode by UPLC-QToF-HRMS.

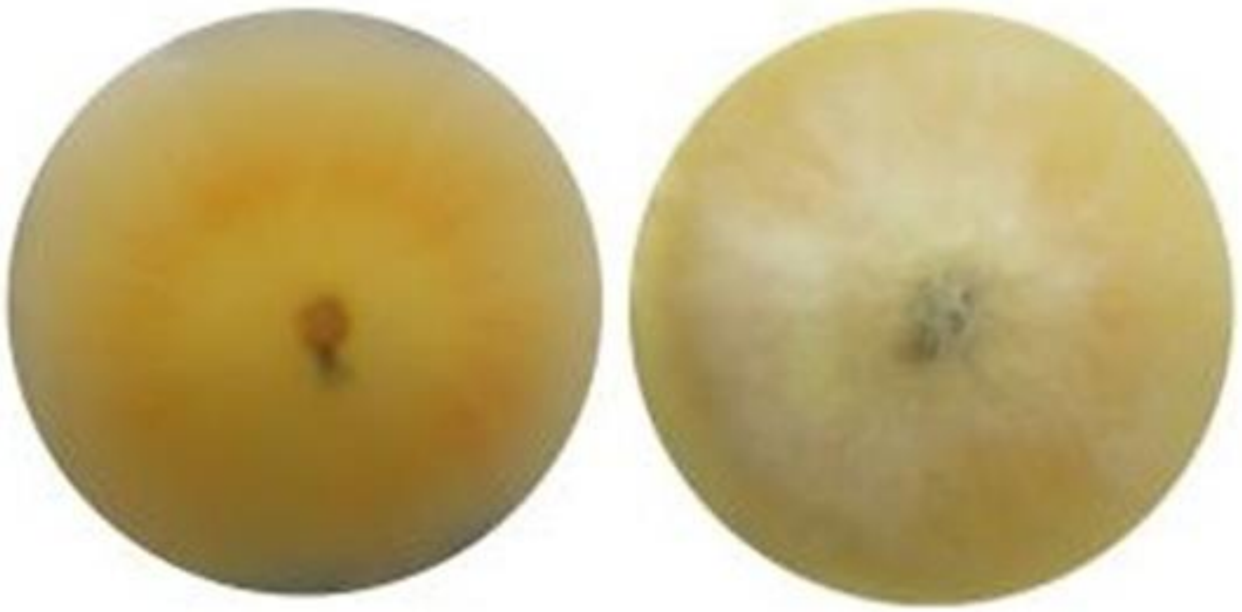

Figure S13. Image of the fungus *Aspergillus terreus* P63 preserved in plate with potato dextrose agar.
